# Highly Luminescent TCNQ in Melamine

**DOI:** 10.1021/acsaom.4c00110

**Published:** 2024-06-06

**Authors:** Vipin Mishra, Arthur Mantel, Peter Kapusta, Alexander Prado-Roller, Hidetsugu Shiozawa

**Affiliations:** † J. Heyrovsky Institute of Physical Chemistry, Czech Academy of Sciences, Prague 182 23, Czech Republic; ‡ Department of Inorganic Chemistry, University of Vienna, Vienna 1090, Austria; § Faculty of Physics, University of Vienna, Vienna 1090, Austria

**Keywords:** TCNQ, DCTC^−^, melamine, fluorescence, quantum yield, DFT

## Abstract

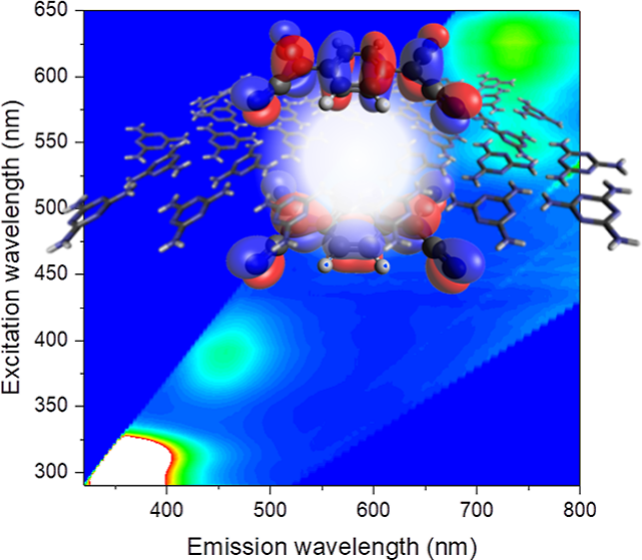

Optical properties of molecules change drastically as a result of interactions with surrounding environments as observed in solutions, clusters, and aggregates. Here, we make 7,7,8,8-tetracyanoquinodimethane (TCNQ) highly luminescent by encapsulating it in crystalline melamine. Colored single crystals are synthesized by slow evaporation of aqueous tetrahydrofuran solutions of melamine and TCNQ. Single-crystal X-ray diffraction reveals the lattice structure of pure melamine, meaning that the color is of impurities. Both mass spectrometry and UV–vis spectroscopy combined with density-functional theory calculations elucidate that the impurity species are neutral TCNQ and its oxidation product, dicyano-*p*-toluoyl cyanide anion (DCTC^–^), whose concentrations in a melamine crystal can be controlled by adjusting the molar ratio between melamine and TCNQ in the precursor solution. Fluorescence excitation–emission wavelength mappings on the precursor solutions illustrate dominant emissions from DCTC^–^ while the emission from TCNQ is quenched by the resonance energy transfer to DCTC^–^. On the contrary, TCNQ in crystalline melamine is a bright fluorophore whose emission wavelength centered at 450 nm with internal quantum yields as high as 19% and slow fluorescence lifetimes of about 2 ns. The method of encapsulating molecules into transparent melamine would make many other molecules fluorescent in solids.

## Introduction

Gemstones exhibit a variety of colors, such as yellow diamond, and some even glow, e.g., fluoresce, phosphoresce, or both, under ultraviolet light. These optical properties often stem from impurities and defects in host crystals.^1^ Luminescent materials with bright emission have recently attracted much interest as they have wide applications,^2−4^ such as emitting layer materials in the light-emitting diodes (LEDs) with various wavelengths. Especially, organic materials are increasingly demanded as organic LEDs, and photovoltaic devices can be thinner, lighter, inexpensive, and more flexible than inorganic ones. Luminescent properties of molecules are largely dependent on the surrounding environment. Most luminescent dyes have π-conjugated planar structures that suffer from quenching due to self-absorption and aggregation in their concentrated solutions and solid states.^5^ This hinders their applications in LEDs that require highly efficient concentrated emitters in solid states. Strategies thus far proposed for brighter solid-state luminescence include the exploration of aggregation-induced emission, and the mitigation of aggregation caused quenching by tailoring molecular stacking or isolating luminophores. The latter could be achieved, for example, by varying functional groups,^6^ cocrystallization,^7^ and encapsulation.^8^

Apparently, naturally nonluminescent materials were out of interest, and hence solid-state effects on their potential luminescence are poorly understood. It is, however, of great importance to explore their potential as luminophores considering an enormous number of nonluminescent organic compounds.

In this article, we demonstrate bright photoluminescence from TCNQ, which hardly luminesces in its solid state, by encapsulating it into crystalline melamine. The photoluminescence quantum yields of TCNQ and anion radical TCNQ^.–^ are typically low due to fast (ps–fs) internal conversion.^9,10^ A better luminescence quantum yield was reported only for TCNQ in some nonpolar solvents^11−13^ and prolonged fluorescence lifetimes for some micro solvation complexes of TCNQ.^14^ These results indicate the significance of intermolecular interactions to the fluorescence quenching in crystalline TCNQ.

Melamine, or 2,4,6-triamino-1,3,5-triazine, is able to withstand strong ultraviolet radiation, which might be a likely reason for its abundance in the primordial soup. Melamine-formaldehyde resins are widely used in a variety of plastic products, such as kitchenware, as well as coating. Neutral melamine, e.g., melamine in water, is colorless and absorbs ultraviolet light^15,16^ and fluoresces only ultraviolet light.^17,18^ It is therefore a perfect host that is transparent in the visible range.

In this work, doped melamine single crystals are synthesized by mixing an aqueous solution of melamine and a tetrahydrofuran (THF) solution of TCNQ, followed by crystallization by vaporization at room temperature. The doping level is tuned by varying the molar ratio of melamine and TCNQ in the aqueous THF. Mass spectrometry combined with UV–vis spectroscopy elucidates that the dopants are TCNQ and dicyano-*p*-toluoyl cyanide (DCTC), an oxidation product of TCNQ. Both TCNQ and DCTC^–^ in melamine are found to be fluorescent in the visible wavelength range with lifetimes in the range of 0.7–5.0 ns for TCNQ and 0.5–2.5 ns for DCTC^–^. Excitation–emission wavelength mapping reveals a dominant emission of TCNQ at low doping levels (molar ratios of TCNQ to melamine in the precursor solution below 0.15%), while at higher doping levels (molar ratios higher than 1%), both TCNQ and DCTC^–^ exhibit strong luminescence. As pure TCNQ is hardly luminescent in its solid state, these results demonstrate that nonradiative relaxations are much reduced by isolating TCNQ in a crystal of melamine. The colorated melamine single crystals are stable as pure melamine, and their luminescence wavelength spans a wide near UV and visible range, which can find various applications in optics and optoelectronics. Our method to encapsulate colorful molecules into crystalline colorless molecules has the potential to make many other nonluminescent organic molecules luminescent.

## Results and Discussion

### Synthesis

Doped melamine crystals have been synthesized by mixing 1 mL of an aqueous solution of melamine (transparent) and 1 mL of a THF solution of TCNQ (olive green). The mixed solution (orange color) slowly evaporates, and crystals precipitate and grow over a few days. Crystals were collected and rinsed with THF for further investigation.

**Figure 1:**
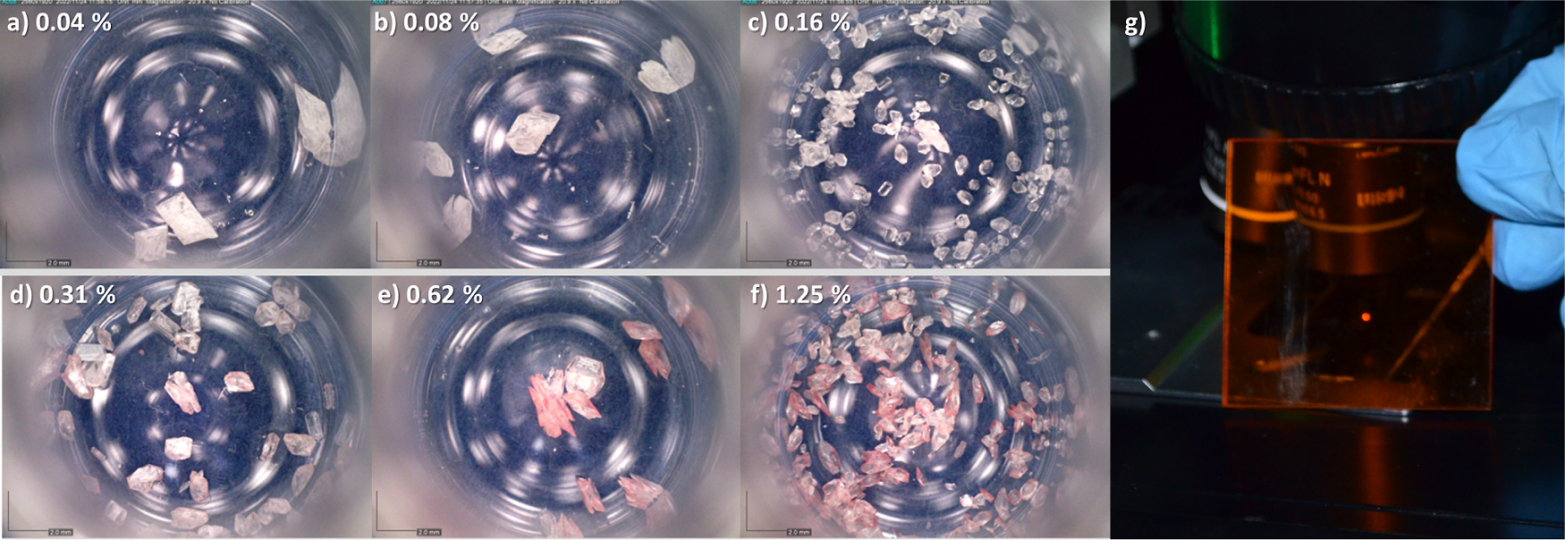
Photographs of crystals of doped melamine (a–f) prepared with mole percentages of TCNQ to melamine of (a) 0.04, (b) 0.08, (c) 0.16, (d) 0.31, (e) 0.62, and (f) 1.25%. Panel (g) shows the photoluminescence from a single crystal on the focus of a green laser viewed through a red filter.

Panels (a–f) in Figure 1 show the photographs of the doped melamine crystals prepared with different molar ratios between melamine and TCNQ (TCNQ, melamine) = (0.04, 100), (0.08, 100), (0.16, 100), (0.31, 100), (0.62, 100), and (1.25, 100), where the numbers in parentheses are the molar concentrations in millimolar (mM) for TCNQ and melamine. The left numbers also correspond to the mole percentages of TCNQ to melamine, namely, 0.04, 0.08, 0.16, 0.31, 0.62, and 1.25%. It is observed that the crystals become colored as the concentration of TCNQ is increased.

### X-ray Crystallography

**Figure 2:**
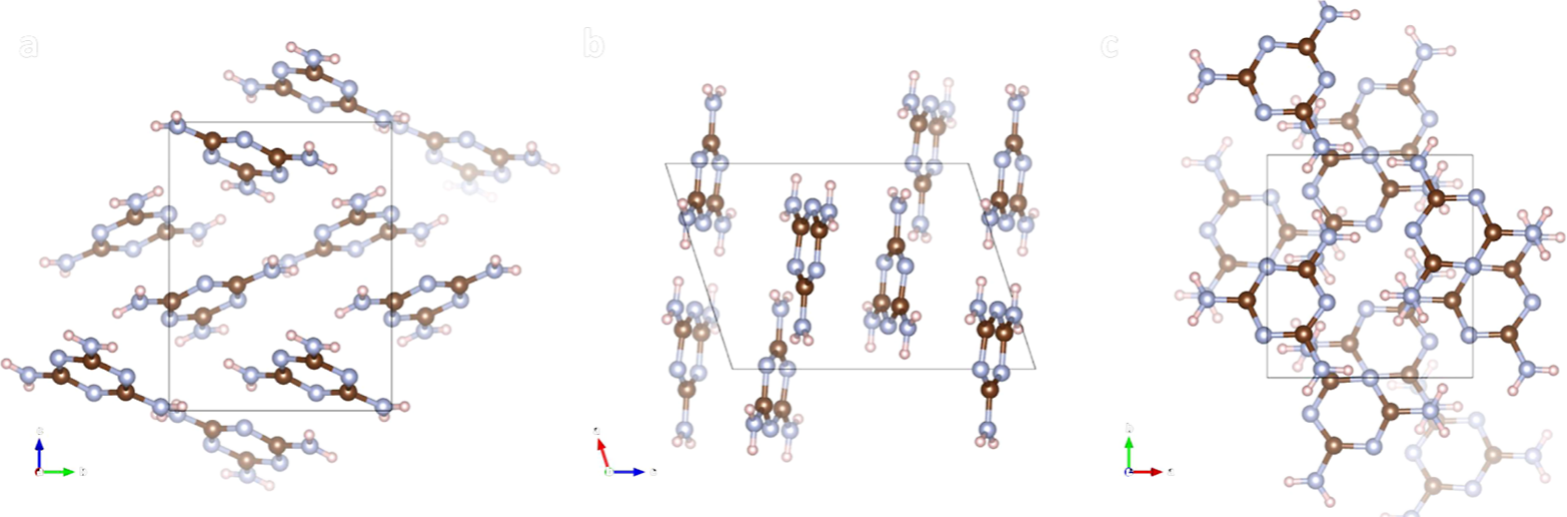
Crystal structure of doped melamine, viewed along axes a, b, and c. The crystal structure is similar to that of melamine.

Single-crystal X-ray diffraction reveals that the lattice structure of the doped melamine, as shown in Figure 2, is similar to that of pure melamine reported in literature.^19^ For more details, see the Supporting Information, section S1. This means that foreign/dopant molecules responsible for the color changes do not crystallize by themselves and remain as a trace of impurity without long-range structural ordering in the crystal of melamine.

### Mass Spectrometry

**Figure 3:**
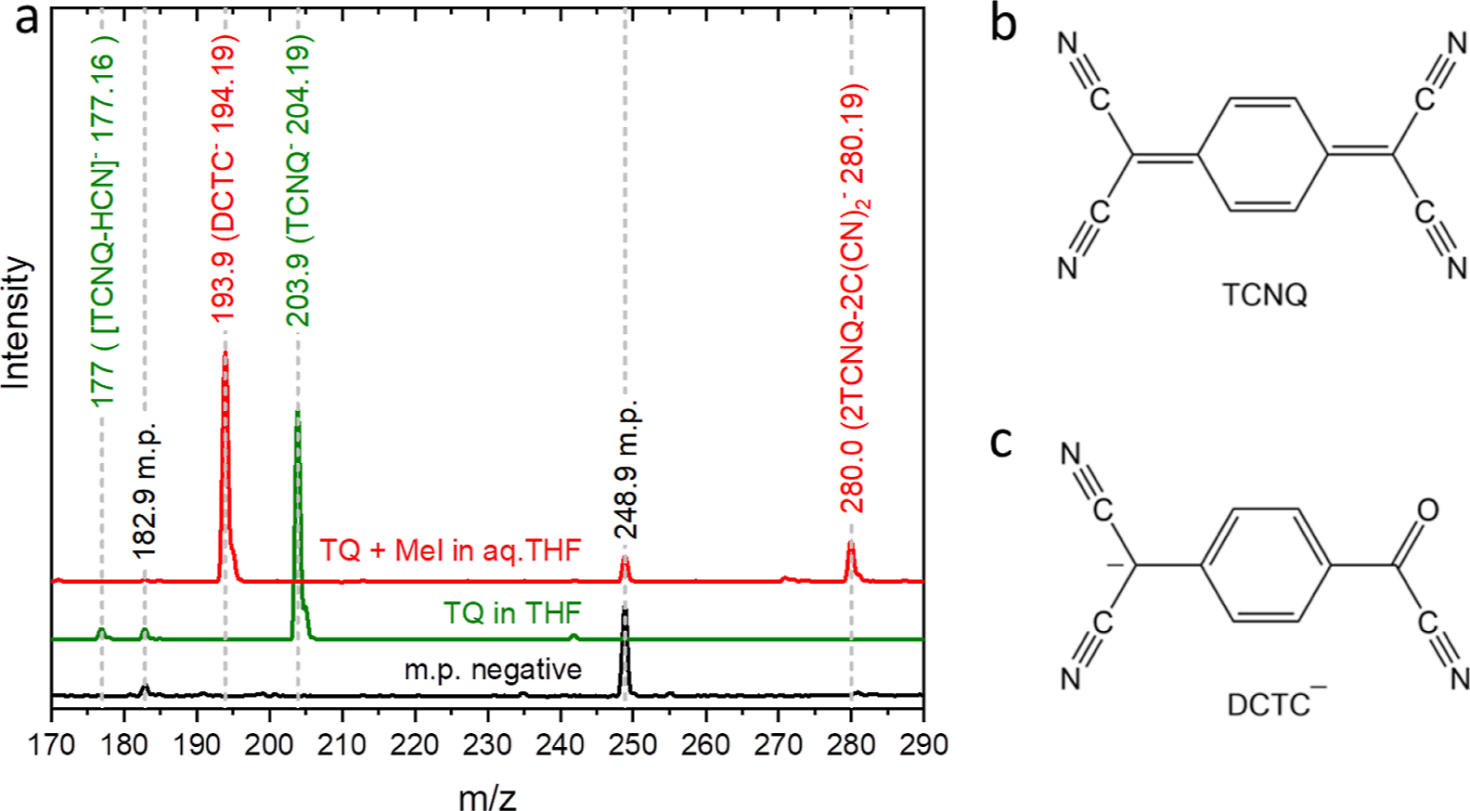
(a) Mass spectra of negative ions for the control mobile phase (m.p.), TCNQ in THF (TQ in THF), TCNQ and melamine with mole percentages of TCNQ to melamine of 1.25% in aqueous THF (TQ + Mel in aq. THF). (b and c) Molecular structures of TCNQ and DCTC^–^.

The nature of dopant species present in the melamine crystal is identified by using high-performance liquid chromatography (HPLC) combined with mass spectrometry (MS). Figure 3a shows the mass spectra for negative ions generated from the control mobile phase (mp), TCNQ in THF (TQ in THF), and TCNQ and melamine (1:1 molar ratio) in aqueous THF (TQ + Mel in aq. THF) by the electrospray ionization (ESI) method. The mass spectra were measured on the output solution of the HPLC for 15 min. The negative ion mass spectrum of TCNQ in THF exhibits the intense peak of TCNQ^–^ at *m*/*z* = ca. 204, the peak of [TCNQ-HCN]^−^^20^ at *m*/*z* = 177, and the peak of contaminants (e.g., at *m*/*z* = 183) present in the mobile phase. The mass spectrum of TCNQ in a mixture of THF and water (1:1 volume ratio) exhibits no peak of TCNQ^–^ and [TCNQ–HCN]^−^, but the peak at *m*/*z* = 194 can be attributed to DCTC^–^. The pink color of melamine crystals precipitated upon slow vaporization of the reaction solution is therefore attributed to DCTC^–^ molecules encapsulated as impurities in melamine crystals. See the Supporting Information, section S2, for the positive-ion mass spectra that exhibit the peaks associated with melamine.

### UV–Vis Absorption

**Figure 4:**
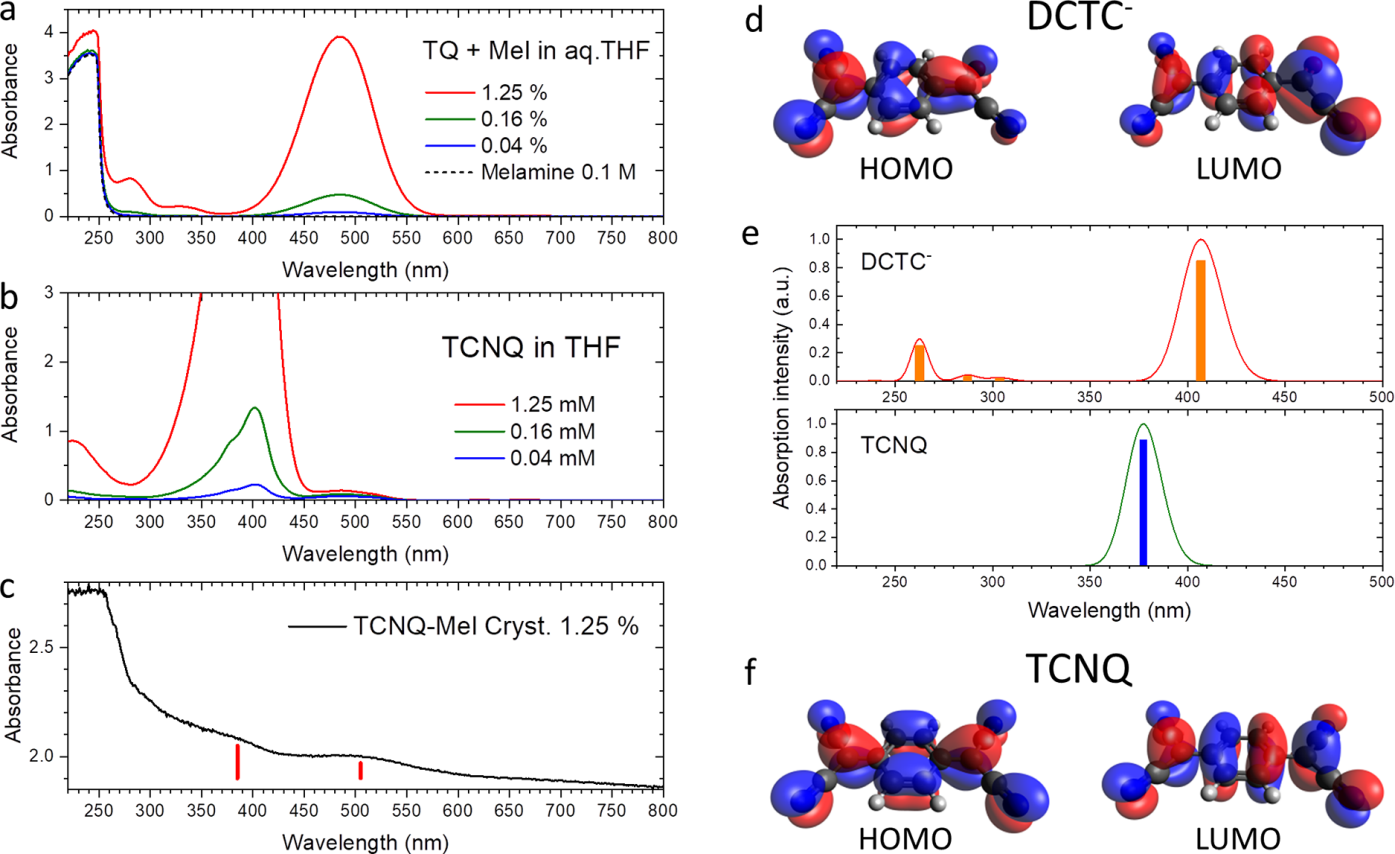
(a) UV–vis spectra for aqueous THF solutions of melamine and TCNQ with mole percentages of TCNQ to melamine of 0.04, 0.16, and 1.25%, in comparison with the spectra for 0.1 M aqueous THF solution of pure melamine. (b) UV–vis spectra for TCNQ in THF with molar concentrations TCNQ of 0.04, 0.16, and 1.25 mM. (c) UV–vis spectrum for a single crystal of doped melamine with a mole percentage of TCNQ to melamine of 1.25%. (d) HOMO and LUMO orbitals of DCTC^–^. (e) Absorption spectra simulated for TCNQ and DCTC^–^ in aqueous THF (for more details, see the Supporting Information, section S4). (f) HOMO and LUMO orbitals of TCNQ.

The concentration of this dopant molecule in the crystal is low (undetectable by single-crystal X-ray diffractometry) and controllable by varying the molar ratio of TCNQ and melamine in the precursor solution, which leads to the color changes as shown in Figure 1. The mass spectrometry reveals the presence of DCTC^-^ in the precursor solutions (i.e., TCNQ in aqueous THF), in which colored melamine crystals are formed. In order to get further insights into the nature of the crystal color, UV–vis spectroscopy has been carried out. Panel (a) in Figure 4 shows the UV–vis spectra for aqueous THF solutions of melamine and TCNQ with mole percentages of TCNQ to melamine of 0.04, 0.16, and 1.25%, in comparison with the spectra for 0.1 M aqueous THF solution of pure melamine. Panel (b) shows the UV−vis spectra for TCNQ in THF with molar concentrations of 0.04, 0.16, and 1.25 mM. All three spectra for TCNQ in THF show the intense visible absorption peak at a wavelength of ∼395 nm. The small peak at 485 nm can be attributed to that of DCTC^–^,^21−23^ possibly due to a trace of water absorbed in THF. For the aqueous THF solutions of TCNQ and melamine, the two peaks of DCTC^–^ located at wavelengths of 282 and 485 nm are dominant, in addition to the strong UV absorption edge of melamine at 250 nm. Figure 4c shows the UV–vis spectrum of a single crystal of doped melamine prepared with a mole percentage of TCNQ to melamine of 1.25%. The absorption edges observed at wavelengths of 385 and 505 nm (indicated by the red vertical bars) can be attributed to TCNQ and DCTC^–^, respectively. In the crystal, both TCNQ and DCTC^–^ seem to be present. Table 1 summaries the absorption wavelengths. See the Supporting Information, section S3 for the UV–vis spectra for aged solutions.

### Density-Functional Theory

Figure 4e shows the UV–vis absorption spectra calculated for DCTC^–^ (top) and TCNQ (bottom) in aqueous THF. For more details, see the Supporting Information, sections S4 and S5. The highest occupied molecular orbital (HOMO) and the lowest unoccupied molecular orbital (LUMO) of DCTC^–^ and those of TCNQ are visualized in panels (d,f), respectively. Although the computed absorption peaks are blue-shifted compared with the experimentally observed peaks, both spectra for the TCNQ and DCTC^–^ reproduce main features observed in the experimental spectra as follows. The computed spectrum for the TCNQ exhibits the single peak at about 377 nm. The computed spectrum for DCTC^–^ exhibits the strongest peak at 408 nm and the weak peak at 263 nm. The major absorption edge for TCNQ is lower than that for the DCTC^–^.

### Photoluminescence

The melamine crystals formed in the aqueous THF solutions of TCNQ and melamine contain both TCNQ and DCTC^–^ as impurity species. Importantly, the doped crystals are strongly luminescent. They are so luminescent that the red emission is visible to the naked eye when a crystal is illuminated by a laser with a wavelength within the 485 nm absorption peak of DCTC^–^. The photograph in Figure 1g shows the photoluminescence on the spot of a 514 nm laser viewed through the long pass filter with a cutoff wavelength of 550 nm (FGL550S, Thorlabs).

**Figure 5:**
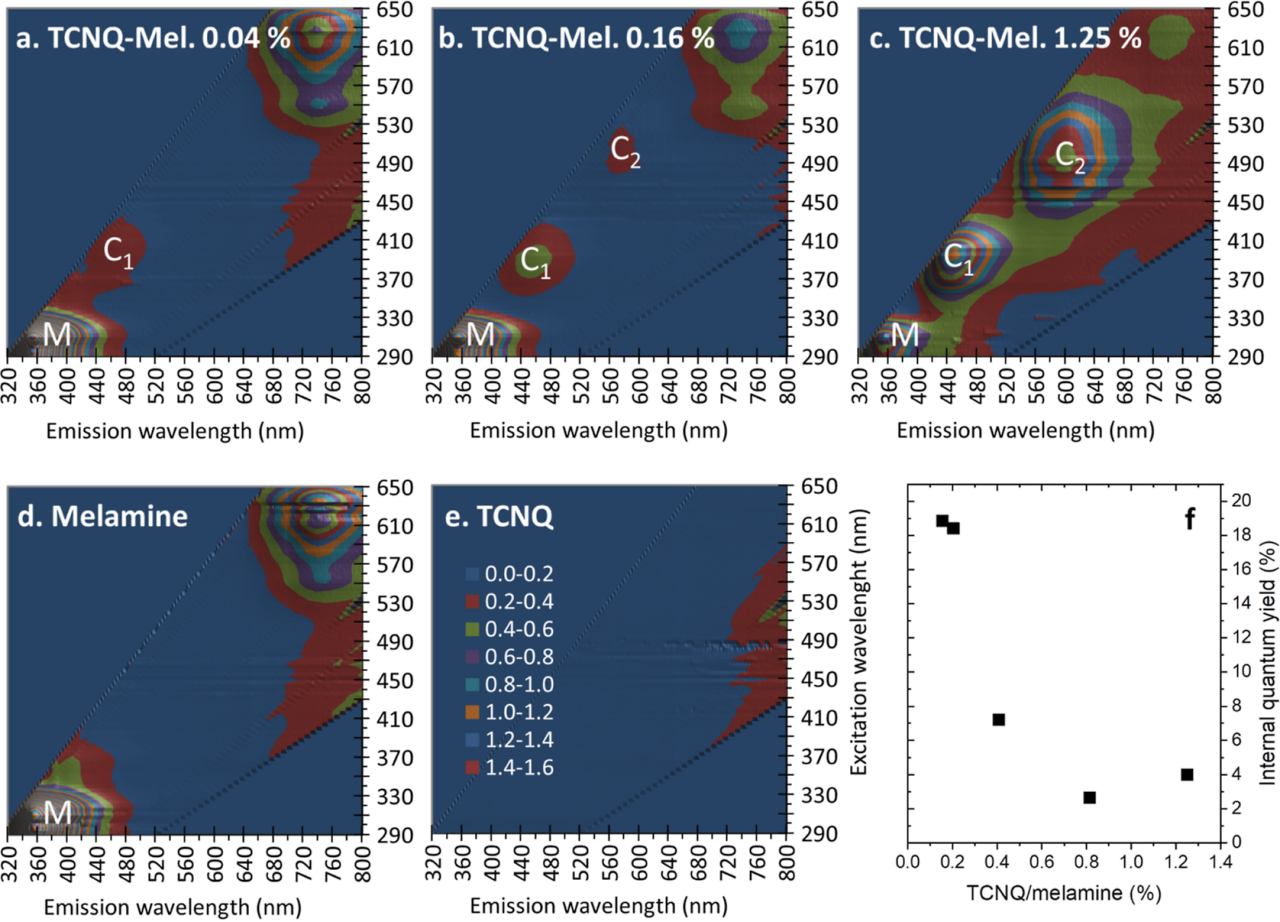
Photoluminescence excitation–emission wavelength maps for single crystals of doped melamine with mole percentages of TCNQ to melamine of (a) 0.04, (b) 0.16, and (c) 1.25%, (d) pure melamine crystal, and (e) powder of TCNQ. (f) Quantum yields of the C_1_ emission for TCNQ-Mel crystals prepared with difference mole percentages of TCNQ to melamine.

**Figure 6:**
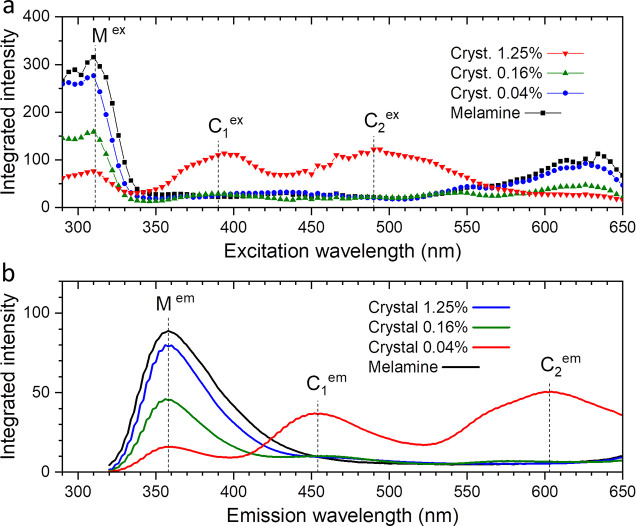
(a) Integrated excitation profiles of the luminescence maps for the single crystals of doped melamine prepared with mole percentages of TCNQ to melamine of 0.04, 0.16, and 1.25%. (b) Integrated emission profiles of the luminescence maps for the single crystals of doped melamine prepared with mole percentages of TCNQ to melamine of 0.04, 0.16, and 1.25%.

Further insights into the luminescence of the doped melamine are gained by mapping the luminescence intensity as functions of both excitation wavelength λ_ex_ and emission wavelength λ_em_ in the UV–visible range on a single crystal. Figure 5 shows the luminescence maps for the doped melamine (TCNQ-Mel) crystals prepared with mole percentages of TCNQ to melamine of 0.04, 0.16, and 1.25% (panels a, b, and c), pure melamine (panel d), and pure TCNQ (panel e). The maps have been normalized by the xenon spectrum and the spectrometer function. In order to compare the maps for the crystals which are different in size, the intensity has been normalized to the intensity of the background line located at . The integrated excitation and emission profiles of the maps are plotted in Figure 6a,b, respectively. All three crystals exhibit the UV luminescence peak of melamine centered at λ_ex_ = 311 nm and λ_em_ = 358 nm, which becomes weaker as the doping concentration is increased. The TCNQ-Mel crystal prepared with 0.04% of TCNQ exhibits the visible emission peak (labeled as C_1_) centered at λ_ex_ ≈ 390 nm and λ_em_ ≈ 454 nm. The C_1_ intensifies as the mole percentage of TCNQ is increased. At 1.25% of TCNQ, the second visible emission peak (labeled as C_2_) discerns at λ_ex_ ≈ 490 nm and λ_em_ ≈ 600 nm. As the excitation and emission wavelengths for both C_1_ and C_2_ coincide with the UV–vis absorption peaks (see panels a, b and c) in Figure 4, the C_1_ and C_2_ peaks can be attributed to the intrinsic emissions from the TCNQ and DCTC^–^, respectively. Crystals of pure TCNQ are basically nonluminescent, as demonstrated in Figure 5e due to its very low quantum yield. The photoluminescence from TCNQ in the precursor solutions is quenched due to the resonance energy transfer to DCTC^–^ (see the Supporting Information, section S6). Immobilized in crystalline melamine, TCNQ becomes highly luminescent, and the resonance energy transfer to DCTC^-^ is evitable, which makes the doped melamine highly luminescent in a wide UV–visible range.

The internal quantum yield of the C_1_ emission was measured using a 405 nm laser. The values estimated for TCNQ-Mel crystals prepared with difference mole percentages of TCNQ to melamine are plotted in Figure 5f. The quantum yields for the TCNQ-Mel crystals at high concentrations of TCNQ (0.81–1.25%) are in the range from 2.6 to 4.9%. This is increased to reach impressive 18.4–18.9% at low concentrations of TCNQ (0.16–0.20%).

### Fluorescence Lifetimes

**Figure 7:**
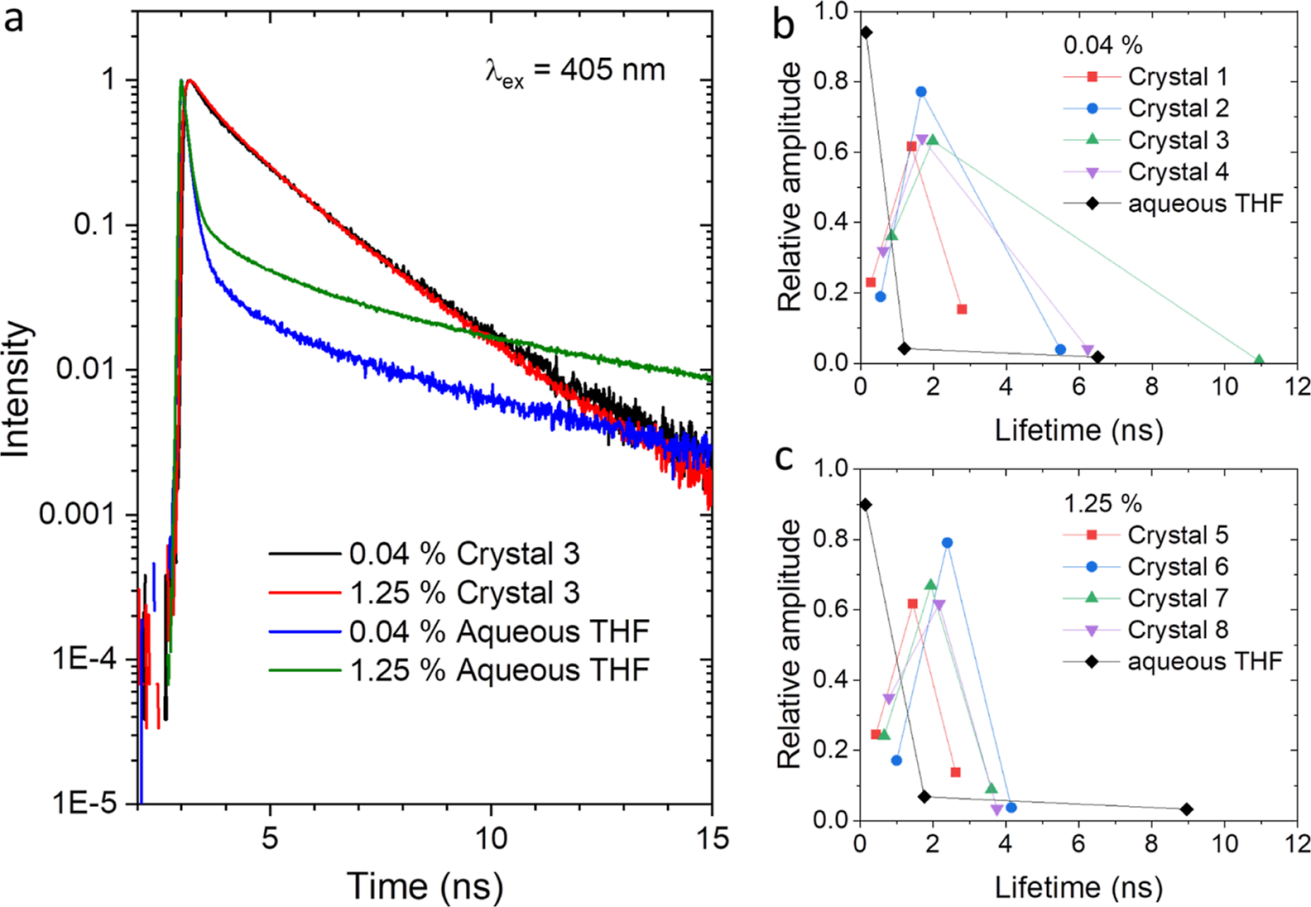
(a) Photoluminescence decay curves for TCNQ (C_1_ peak) in single crystals of doped melamine prepared with mole percentages of TCNQ to melamine of 0.04 and 1.25%, in comparison with those for aqueous THF solutions of melamine and TCNQ with mole percentages of TCNQ to melamine of 0.04 and 1.25%, measured at a laser wavelength of 405 nm and an emission band of 425–455 nm. (b) Three lifetimes and their relative amplitudes evaluated for 0.04% crystals 1, 2, 3, and 4 and for the 0.04% precursor solution. (c) Three lifetimes and their relative amplitudes evaluated for 1.25% crystals 5, 6, 7, and 8 and for the 1.25% precursor solution.

Further insights into the environmental factors are gained from the photoluminescence lifetimes. TCNQ (C_1_ peak) has been resonantly excited at 405 nm. See the results for DCTC^–^ (C_2_ peak) measured at 470 nm in Supporting Information, section S7. Figure 7a shows the decay curves for TCNQ (C_1_) in single crystals of doped melamine prepared with mole percentages of TCNQ to melamine of 0.04% (crystal 1) and 1.25% (crystal 5), and the decay curves for the respective precursor aqueous THF solutions, measured at a laser wavelength of 405 nm with an emission band of 425–455 nm. All of the curves can be fitted with triple exponential functions. Lifetimes and relative amplitudes evaluated for different 0.04% crystals are plotted in panel (b) and for 1.25% crystals in panel (c). In both 0.04 and 1.25% crystals, dominant fluorescence lifetimes are approximately 2 ns, which is much slower than about 0.14 ns estimated for the precursor solutions. The much delayed fluorescence is apparently because of the solid-state environment in which fluorescent molecules are immobilized in a crystal of melamine, and the passivation of nonradiative decay processes, e.g., fast (ps–fs) internal conversion,^9,10^ allows TCNQ to luminesce.

## Conclusions

We have demonstrated that nonluminous TCNQ becomes highly luminescent in crystals of melamine. The method of encapsulating colorful guest molecules in a transparent, large-gap host material can be utilized for many other host–guest combinations. This opens up new avenues of exploration for advanced optical materials.

## Experimental Section

### Materials

Melamine (purity >99%) and tetracyanoquinodimethane (TCNQ, purity >99%) were purchased from Merck and used as supplied. THF anhydrous was purchased from Thermo scientific (cat. no. 11319917), and freshly filtered deionized water was used as the solvent for synthesis. Methanol purchased from Penta Chemicals (catalog no. 21240-11000) was used for washing doped melamine crystals.

### Synthesis

0.0253 g portion of melamine was mixed with 1 mL of deionized water (0.2 M equivalent) and 1 mL of a THF solution of TCNQ (a molar concentration of 0.08, 0.16, 0.32, 0.62, 1.24, or 2.50 mM). The mixed solution in a closed 5 mL glass vial was heated at 80 °C for 5 min until all melamine crystals were completely dissolved, then cooled down to room temperature. The solution was left in an open glass vial at room temperature for 48 to 72 h, which resulted in the slow evaporation of the solvents and the formation of doped crystals of melamine. The crystals were collected and rinsed first with methanol multiple times, then with THF, and kept dry or in THF.

**Table 1:** Absorption Wavelengths Observed for TCNQ-Mel in aq. THF, TCNQ in THF, and TCNQ-Mel Cryst. 1.25% and Computed for TCNQ and DCTC^–^

	Exp. (nm)			DFT (nm)	
samples	TCNQ-Mel in aq. THF	TCNQ in THF	TCNQ-Mel Cryst. 1.25%	TCNQ	DCTC^–^
	282				263
		395	385	377	
	485	485	505		408

### Solubility

The solubilities of doped melamine crystals in water and different organic solvents have been studied. The results are summarized in Table 2. The crystals are soluble in DMF, marginally in water, and sparingly soluble in methanol, while they remain nearly insoluble in acetonitrile, acetone, ethyl acetate, and THF. This solubility trend of the doped crystal is similar to that of pure melamine.

**Table 2:** Solubility of Doped Melamine Crystals in Water and Organic Solvents

solvent	solubility of doped crystal
DMF	soluble
water	marginally soluble
methanol	sparingly soluble
acetonitrile	insoluble
acetone	insoluble
ethyl acetate	insoluble
THF	insoluble

### X-ray Analysis

The X-ray intensity data were measured on a Bruker D8 Venture diffractometer equipped with a multilayer monochromator, Mo K/α INCOATEC micro focus sealed tube, and Oxford cooling systems. The structure was solved by Direct methods. Non-hydrogen atoms were refined with anisotropic displacement parameters. Hydrogen atoms were inserted at calculated positions and refined with the riding model. The following software was used: Bruker SAINT software package^24^ using a narrow-frame algorithm for frame integration, SADABS^25^ for absorption correction, OLEX2^26^ for structure solution, refinement, molecular diagrams, and graphical user-interface, ShelXle^27^ for refinement and graphical user-interface SHELXS-2015^28^ for structure solution, SHELXL-2015^29^ for refinement, and Platon^30^ for symmetry check.

### Mass Spectrometry

Sample solutions of the doped melamine for the HPLC-CMS were prepared in deionized water. Acetonitrile–water mixture of various volume ratios (see Table 3) was used as the mobile phase to run the HPLC-CMS experiments. Integrated mass spectrometry (Advion expression CMS) with HPLC was used to record the mass spectra for both positive and negative ions generated by ESI. The HPLC-CMS data of the mobile phase (mp) were also recorded to ascertain the preliminary peaks already present before loading the sample solutions.

**Table 3:** Water–to–Acetonitrile Volume Ratios during the HPLC-CMS Measurements

duration (min.)	water (%)	acetonitrile (%)
0.50	95.0	5.0
10.00	5.0	95.0
12.50	5.0	95.0
12.51	50.0	50.0

### Photoluminescence Spectroscopy

Fluorescence excitation and emission wavelength maps were measured on a crystal in a quartz cuvette (Ossila, C2003P1) using Horiba Fluorolog-3 equipped with a 450 W ozone-free xenon short-arc and R928P photomultiplier tube with a DM302 PC Acquisition Module. For both excitation and emission monochromators, diffraction gratings with a line density of 1200 L/mm and a blaze wavelength of 500 nm were used, and the entrance and exit slits were set to bandpasses of 2, 3, or 4 nm, depending on the fluorescence intensity.

### Fluorescence Lifetime and Autocorrelation Measurements

Fluorescence lifetime imaging microscopy was carried out using an Olympus IX71 inverted microscope equipped with PicoQuant HydraHarp 400 time-correlated single photon counting electronics, and MicroPhotonDevices single-photon avalanche diode. Experiments were performed at an excitation laser wavelength of 405 nm using PicoQuant LDH-D-C-400B laser, Semrock 390/40 nm BrightLine single-band bandpass filter, Semrock R405 dichroic filter, and HQ440/30M emission filter and at 470 nm using PicoQuant LDH-P-C-470 laser, Chroma Z470/635RPC dichroic filter, and HQ525/50M emission filter.

### Density-Functional Theory

The density-functional theory (DFT) calculations were carried out using the ORCA quantum chemistry program package, version 5.0.4. B3LYP hybrid functional and DEF2-SVP basis set were used. The transition electric dipole moments were computed based on the time-dependent DFT. The SCF convergence criteria were set to TightSCF (energy change 1.0 × 10^–8^ au). Solvent effects were taken into account using the conductor-like polarizable continuum model, in which the bulk solvent was treated as a conductor-like polarizable continuum with the refractive index and the dielectric constant of the medium.^31^ A refractive index of 1.3749 and a dielectric constant of 43.4998 were set for a 1:1 mixture (by volume) of water and THF.^32^ The absorption spectra were obtained by convoluting the transition rates with a Gaussian function (a full width at half-maximum of 1500 cm^–1^).

## Supporting Information Available

X-ray analysis; positive-ion mass spectra; UV–vis spectroscopy on aged solutions; solvent effects in DFT calculations; DFT calculations of dimers; fluorescence excitation–emission wavelength maps for the precursor solutions; and fluorescence lifetime for DCTC^–^ (C_2_ peak) (PDF)
