# Supplementary material for: Highly Luminescent TCNQ in Melamine
Source: ACS Appl Opt Mater. 2024 Jun 6;2(6):1128–35. doi: 10.1021/acsaom.4c00110 (PMC11217937; doi:10.1021/acsaom.4c00110)
Supplement: Supplementary file 1 — ot4c00110_si_001.pdf [file ot4c00110_si_001.pdf]

# Supporting Information (SI)

## Highly luminescent TCNQ in melamine

June 5, 2024

**Vipin Mishra,**[1] **Arthur Mantel,**[1] **Peter Kapusta,**[1] **Alexander Prado-Roller,**[2] **and Hidetsugu Shiozawa**[1,3*]

[1]J. Heyrovsky Institute of Physical Chemistry, Czech Academy of Sciences, Prague, 182 23, Czech Republic

[2]Department of Inorganic Chemistry, University of Vienna, Vienna, 1090, Austria

[3]Faculty of Physics, University of Vienna, Vienna, 1090, Austria

[*]To whom correspondence should be addressed; E-mail: hide.shiozawa@jh-inst.cas.cz & hidetsugu.shiozawa@univie.ac.at

# S1 X-ray analysis

Crystal data, data collection parameters, and structure refinement details are given in Table S1. Asymmetric Unit visualized in Figure S1. Figure S2 is a packing view and show Hydrogen bond interactions. Figures S3 to S5 support information about the data quality.

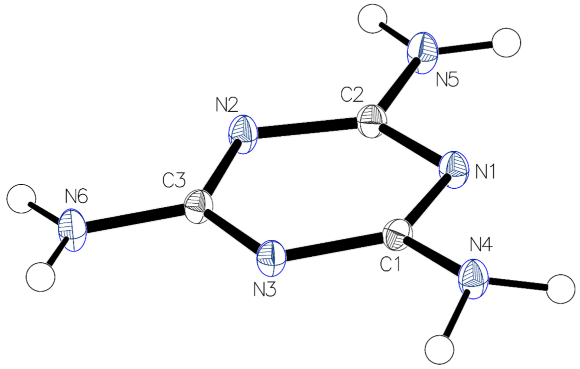


Figure S1: Asymmetric Unit of drawn with 50% displacement ellipsoid. The bond precision for C-C single bonds is 0.0016 Å. N4 shows tetrahedron geometry and the rest densities related to the positions of the Hydrogens and is not planar as N5 and N6. This is in good accordance to the different bond length of C1-N4 to C2-N5 and C3-N6. For detailed data please open the cif code. Hydrogenbonds are discussed in Figure 2.

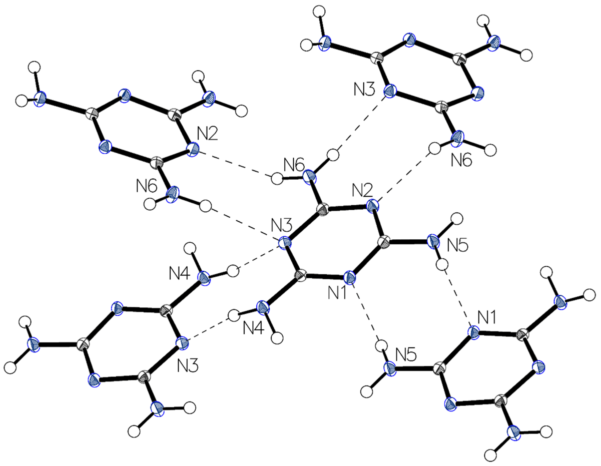


Figure S2: Three different types of H-Bond are formed pairwise. The first type can be sorted with N6H-N3 & N2-HN6. The second is N5H-N1 and the third N4H-N3. The three types alwas form couples of the target molecule. Two Hydrogen atoms show almost no interaction to neighboring Nitrogens, one located at N4 and one at N5. For detailed data please open the cif code.

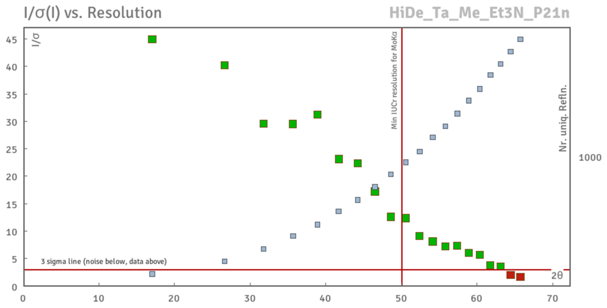


Figure S3: Data quality I: All data are above the three sigma line along the min IUCR definition.

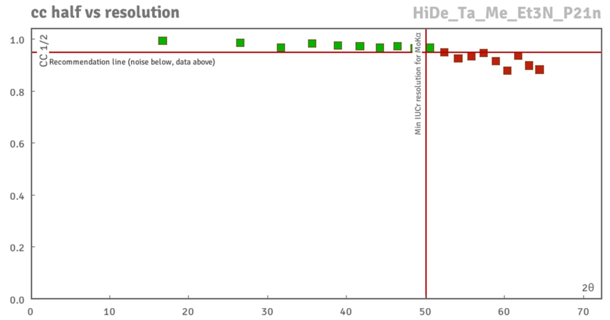


Figure S4: Data quality II: All data are above the recommendation line along the min IUCR definition.

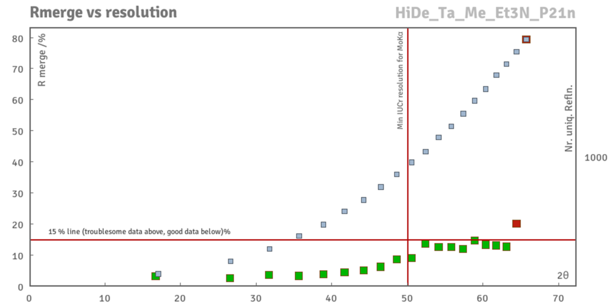


Figure S5: Data quality III: All data are below the recommendation 15% $R_{merge}$ line along the min IUCR definition.

| | |
|---|---|
| Identification code | HiDe_Ta_Me_Et3N_P21n |
| Empirical formula | C3H6N6 |
| Formula weight | 126.14 |
| Temperature/K | 125.0 |
| Crystal system | monoclinic |
| Space group | $P2_1/n$ |
| a/Å | 7.2496(3) |
| b/Å | 7.4597(3) |
| c/Å | 10.1584(3) |
| $\alpha/^\circ$ Å | 90 |
| $\beta/^\circ$ Å | 108.1080(19) |
| $\gamma/^\circ$ Å | 90 |
| Volume/$Å^3$ | 522.16(3) |
| Z | 4 |
| $\rho_{calc}$g/cm$^3$ | 1.605 |
| $\mu$/mm$^{-1}$ | 0.119 |
| F(000) | 264.0 |
| Crystal size/mm$^3$ | 0.396 × 0.34 × 0.014 |
| Radiation | MoK$\alpha$ ($\lambda$ = 0.71073) |
| 2$\Theta$ range for data collection/° | 6.104 to 60.042 |
| Index ranges | $-10 \leq h \leq 9$, $-10 \leq k \leq 10$, $-14 \leq l \leq 14$ |
| Reflections collected | 7444 |
| Independent reflections | 1519 [$R_{int}$ = 0.0431, $R_{sigma}$ = 0.0347] |
| Data/restraints/parameters | 1519/2/90 |
| Goodness-of-fit on $F^2$ | 1.107 |
| Final R indexes [I>=2$\sigma$ (I)] | $R_1$ = 0.0424, w$R_2$ = 0.1149 |
| Final R indexes [all data] | $R_1$ = 0.0571, w$R_2$ = 0.1211 |
| Largest diff. peak/hole / eÅ$^{-3}$ | 0.28/-0.36 |
| Detector distance/mm | 36 |
| Time/Frame /s | 1 |
| #Frames | 1000 |
| Frame width | 0.360 |

Table S1: Sample and crystal data, Data collection and structure refinement

## S2 Positive ion mass spectra

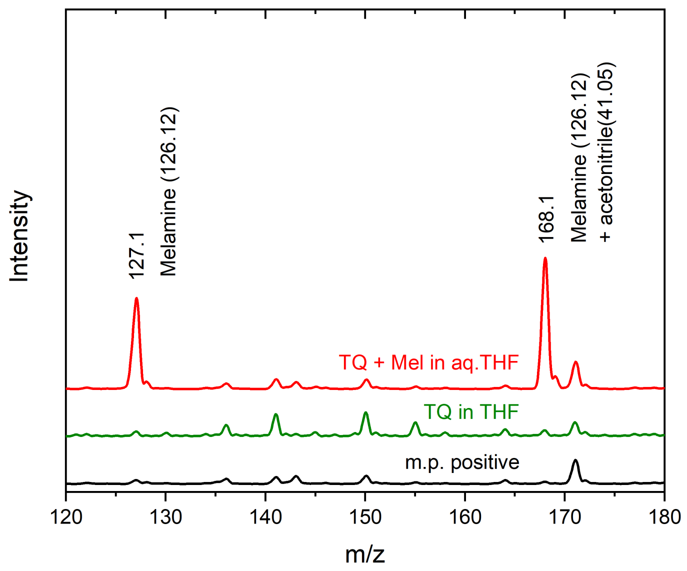


Figure S6: Mass spectra of positive ions for the control mobile phase (m.p. positive), TCNQ in THF (TQ in THF), TCNQ and melamine (1:1 molar ratio) in aqueous THF (TQ + Mel in aq. THF).

The positive ion mass spectrum of the TCNQ-melamine in Fig. S6 shows peaks at m/z (mass divided by charge number) = 127.1 and 168.1 which correspond to melamine (melamine + $H^+$) and an adduct of melamine and acetonitrile (Melamine + $CH_3CN$ + $H^+$), respectively. Other peaks like those at m/z 42.3, 83.2, 101.2, 143.2 are present in the spectrum for the control mobile phase (m.p. positive).

# S3 UV-Vis spectroscopy on aged solutions

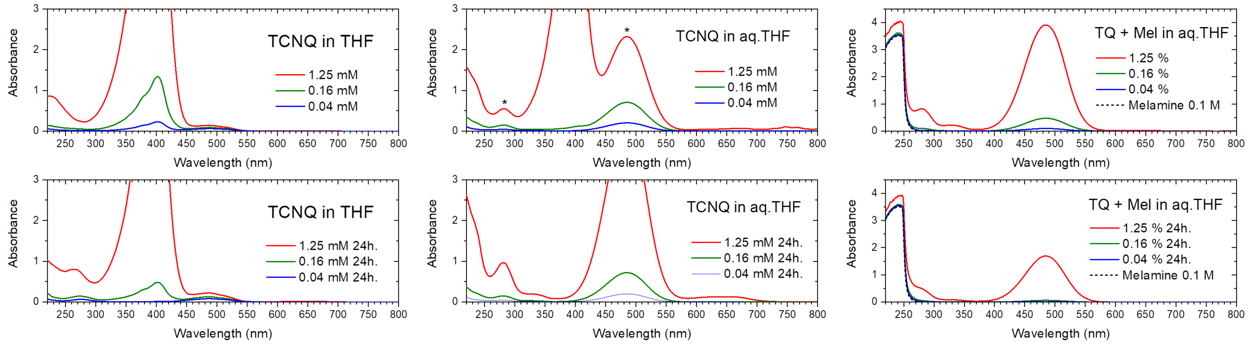


Figure S7: (left) UV-Vis spectra for aqueous solutions of TCNQ with molar concentrations of 0.04, 0.16 and 1.25 mM, as freshly prepared (top) and after 24 hours (bottom). (middle) UV-Vis spectra for aqueous THF solutions (1:1 volume ratio) of TCNQ with molar concentrations of 0.04, 0.16 and 1.25 mM, as freshly prepared (top) and after 24 hours (bottom). (right) UV-Vis spectra for aqueous THF solutions of TCNQ and melamine with mole percentages of TCNQ to melamine of 0.04, 0.16 and 1.25 %, as freshly prepared (top) and after 24 hours (bottom).

Figures S7 left shows the UV-Vis spectra of TCNQ in THF with molar concentrations of 0.04, 0.16 and 1.25 mM, as freshly prepared (top) and after 24 hours (bottom). It is observed that a small portion of TCNQ has been transformed to $DCTC^-$.

Figures S7 middle shows the UV-Vis spectra of TCNQ freshly dissolved in aqueous THF (1:1 volume ratio) with molar concentrations of 1.25, 0.16 and 0.04 mM, as freshly prepared (top) and after 24 hours (bottom). The TCNQ peak becomes dominant as the concentration is increased. This demonstrates that the molar ratio between TCNQ and $DCTC^-$ in aqueous THF is adjusted from almost pure $DCTC^-$ to dominantly TCNQ by varying the concentration from 0.04 mM to 1.25 mM. For the freshly prepared solution (top), the intense visible absorption peak of TCNQ at a wavelength of $\sim$ 395 nm and the two peaks of $DCTC^-$ at wavelengths of 282 and 485 nm, marked with stars, coexist. Interestingly, the molar ratio between TCNQ and DCTC in aqueous THF changes from almost pure $DCTC^-$ to dominantly TCNQ by varing the concentration from 0.04 mM to 1.25 mM. After 24 hours

(bottom), TCNQ has entirely been transformed into $DCTC^-$.

Figures S7 right shows the UV-Vis spectra of TCNQ and melamine in aqueous THF with mole percentages of TCNQ to melamine of 0.04, 0.16 and 1.25 %, as freshly prepared (top) and after 24 hours (bottom). The spectra demonstrate that the solutions contain only $DCTC^-$ as freshly prepared. These solutions were used for the crystallisation reported in the manuscript. After 24 hours, the peaks of $DCTC^-$ have become weaker. This indicates that the $DCTC^-$ decomposes slowly in aqueous THF.

# S4 Solvent effects in DFT calculations

Figure S8 compares the absorption spectra computed for $DCTC^-$ in vacuum and that in aqueous THF. The spectra were obtained by convoluting the absorption lines with a Gaussian function (a FWHM of 1500 $cm^{-1}$). The Conductor-like Polarizable Continuum Model (C-PCM) was used to take the effect of solvents into account.[1] For the aqueous THF solvent (1:1 volume ratio), a dielectric constant of 43.4998 and the refractive index of 1.3749 were derived from interpolation of data in Ref.[2]

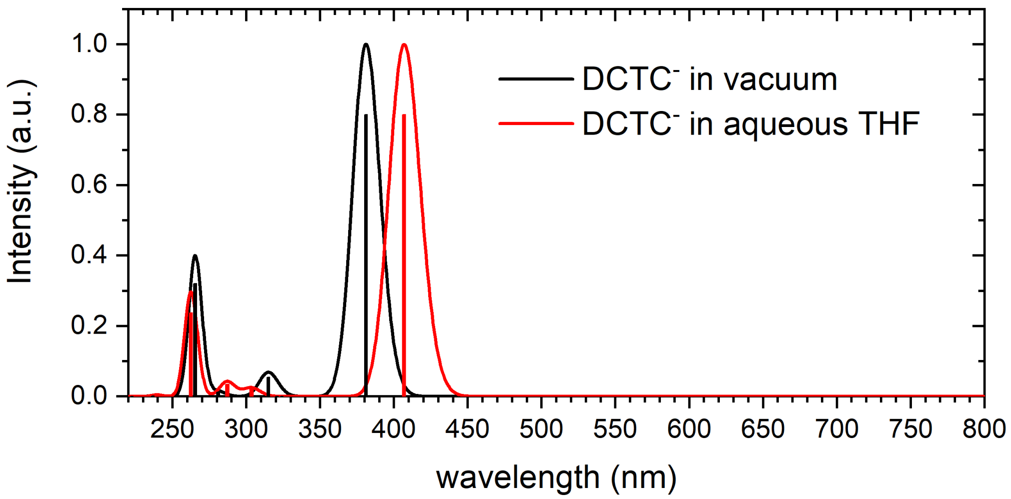


Figure S8: Absorption spectra computed for $DCTC^-$ in vacuum and that in aqueous THF.

# S5 DFT calculations of dimers

The optimized structure and calculated UV-Vis spectra of possible charge transfer complexes, melamine-TCNQ, and melamine-$DCTC^-$ in aqueous THF are shown in Figure S9. Note that TCNQ dimer and $DCTC^-$ dimer were found to be instable as the molecules are far apart in the optimized structure so that the UV-Vis spectrum of the dimer is very similar to the monomer's spectrum. In Fig. S9a, the UV-Vis spectra of the melamine-TCNQ and melamine-$DCTC^-$ are plotted in comparison with the spectra of melamine, TCNQ and $DCTC^-$. Melamine has no absorption in the plotted range. The effect of the pairing is minor.

The major absorption peak in the visible range is only slightly red-shifted in both cases. In other words, the interactions between the dopant and melamine are weak. It justifies our claim that neutral TCNQ or DCTC$^-$ molecules are encapsulated in melamine.

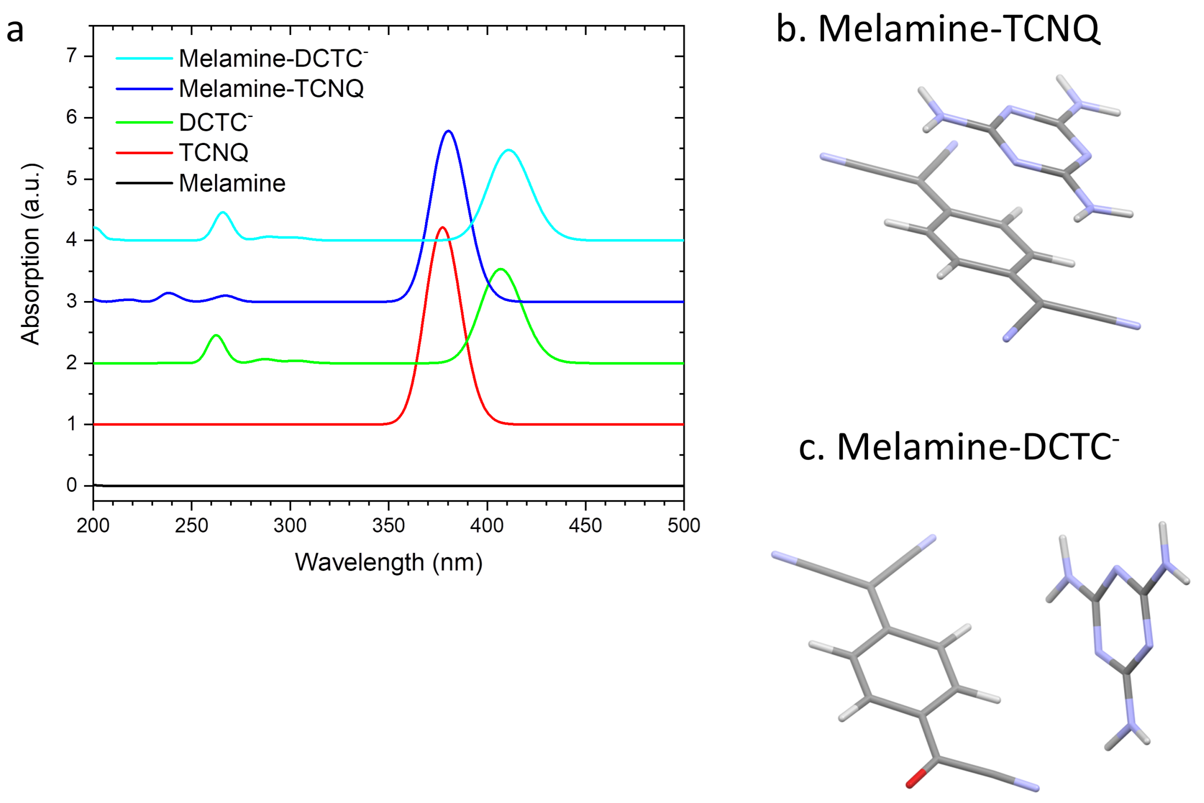


Figure S9: a) Absorption spectra calculated for melamine-TCNQ dimer and melamine-DCTC$^-$ dimer, in comparison with the spectra for melamine, TCNQ and DCTC$^-$. b) Optimized structure of melamine-TCNQ dimer. c) Optimized structure of melamine-DCTC$^-$ dimer.

# S6 Fluorescence excitation-emission wavelength maps for the precursor solutions

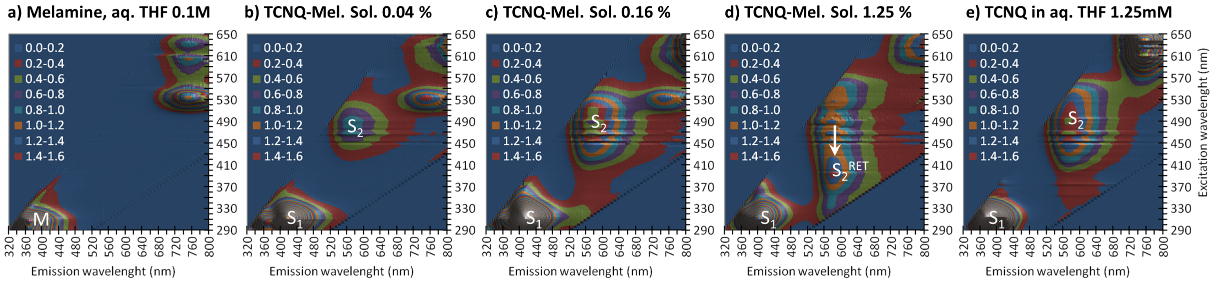


Figure S10: Photoluminescence excitation-emission wavelength maps for (a) 0.1 M aqueous solution of pure melamine, aqueous THF solutions of melamine and TCNQ with mole percentages of TCNQ to melamine of (b) 0.04 %, (c) 0.16 % and (d) 1.25 %, and (e) 1.25 mM aqueous THF solution of TCNQ.

The luminescence maps for the precursor aqueous THF solutions of melamine and TCNQ prepared with mole percentages of TCNQ to melamine of 0.04, 0.16 and 1.25 % are shown in Fig. S10b, c and d, respectively. The maps have been normalized by the Xenon spectrum and the grating function. The integrated excitation and emission profiles of the maps are plotted in symbols and lines, respectively, in Fig. S11.

The map for pure melamine in aqueous THF (panel a) exhibits the UV luminescence peak (labeled as M) centered at $\lambda_{ex}$ = 306 nm and $\lambda_{em}$ = 368 nm. For the solutions of melamine and TCNQ (panels b, c and d), this peak is overwhelmed by the more intense UV-Vis emission peak (labeled at $S_1$) at $\lambda_{ex}$ = 311 nm and $\lambda_{em}$ = 415 nm (at 0.04 %). In addition, another visible emission (labeled as $S_2$) is observed at $\lambda_{ex} \sim 480$ nm and $\lambda_{em}$ = 575 nm (at 0.04 %). As the mole percentage of TCNQ is increased, the $S_1$ emission wavelength is shifted to lower wavelengths, while the excitation wavelength is only slightly red-shifted (see Fig. S11). The intensity becomes greater as the mole percentage of TCNQ is increased from 0.04 to 0.16 %, then at 1.25 % it becomes weaker again. In turn, the $S_2$ emission continues to intensify and get slightly red-shifted as the mole percentage of TCNQ is increased. At 1.25 % the $S_2$ emission is elongated to lower excitation wavelengths (indicated by the down

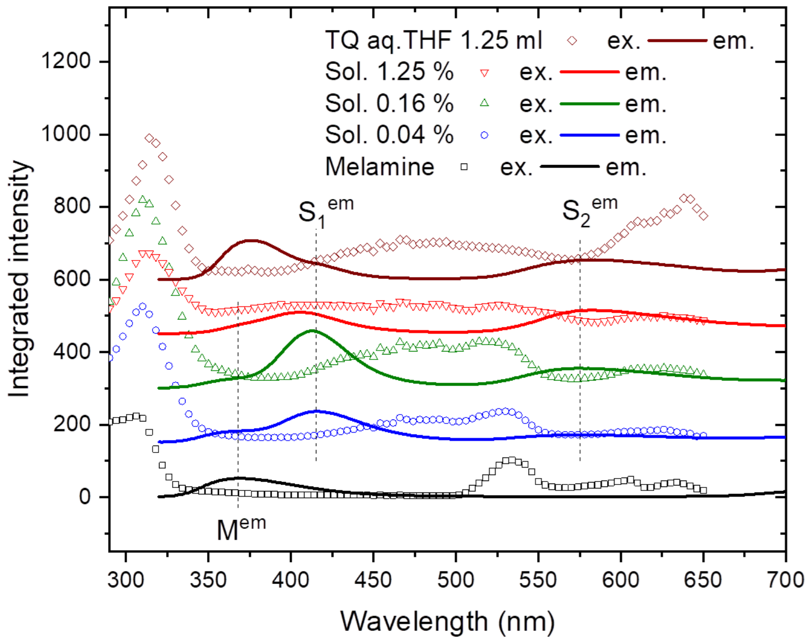


Figure S11: Integrated excitation (ex.) and emission (em.) profiles of the luminescence maps for the aqueous THF solutions of melamine and TCNQ with mole percentages of TCNQ to melamine of 0.04, 0.16 and 1.25 %. The corresponding profiles for the melamine solution are also plotted.

arrow) to exhibit the highest emission at $\lambda_{ex} \sim 390$ nm (labeled as $S_2^{RET}$), while the emission wavelength is unchanged. These vertical shifts can be understood as results of resonance energy transfer processes through which some portion of photo-excited excitons relax to the lowest bound state responsible for the $S_2$ emission. The excitation energy for the $S_2$ peak corresponds well to the UV-Vis absorption peak of $DCTC^-$. Hence, the $S_2$ peak can be attributed to the photoluminescence from the $DCTC^-$. $\lambda_{ex} \sim 390$ nm for the downshifted $S_2^{RET}$ at 1.25 % (panel d) matches the UV-Vis absorption wavelength for TCNQ in THF. This indicates some of TCNQ remain unoxidized in the solution, and the resonance energy transfer occurs from TCNQ to $DCTC^-$.

Both $S_1$ and $S_2$ peaks are also observed for a freshly prepared aqueous THF solution of TCNQ without melamine (Fig. S12c). After 24 hours, the $S_2$ peak of $DCTC^-$ disappears and the $S_1$ peak intensifies (see panels c and d in Fig. S12). This indicates that the $S_1$ emission

originates from a decomposed product of $DCTC^-$.

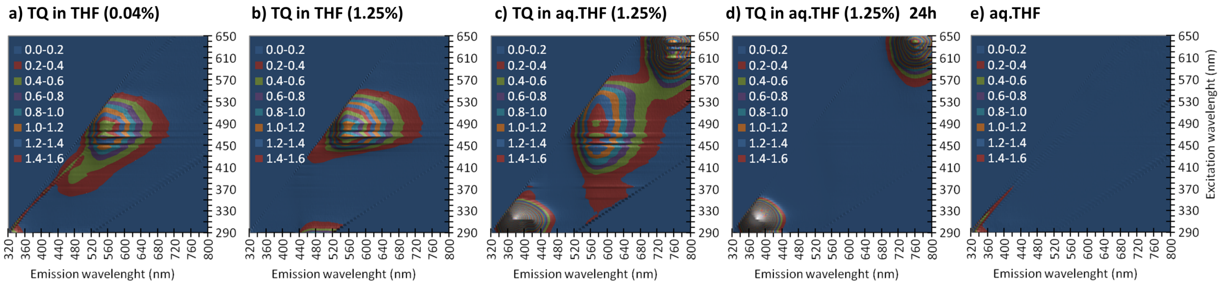


Figure S12: Photoluminescence excitation-emission wavelength maps for THF solutions of TCNQ with mole percentages of TCNQ to melamine of (a) 0.04 % and (b) 1.25 %, aqueous THF solution with 1.25 % (c) as freshly prepared and (d) after 24 hours, and (e) THF.

## S7 Fluorescence lifetime for $DCTC^-$ ($C_2$ peak)

Figure S13a shows the decay curves for $DCTC^-$ ($C_2$) in the single crystals of doped melamine prepared with mole percentages of TCNQ to melamine of 0.04 % (Crystal 1) and 1.25 % (Crystal 8), and the decay curves for the respective precursor aqueous THF solutions, measured at a laser wavelength of 470 nm and an emission band of 500 – 550 nm. All the curves can be fit with triple exponential functions. Lifetimes and relative amplitudes evaluated for different 0.04 % crystals are plotted in panel b, and for 1.25 % crystals in panel c. In both 0.04 % and 1.25 % crystals, dominant fluorescence lifetimes are approximately 2 ns.

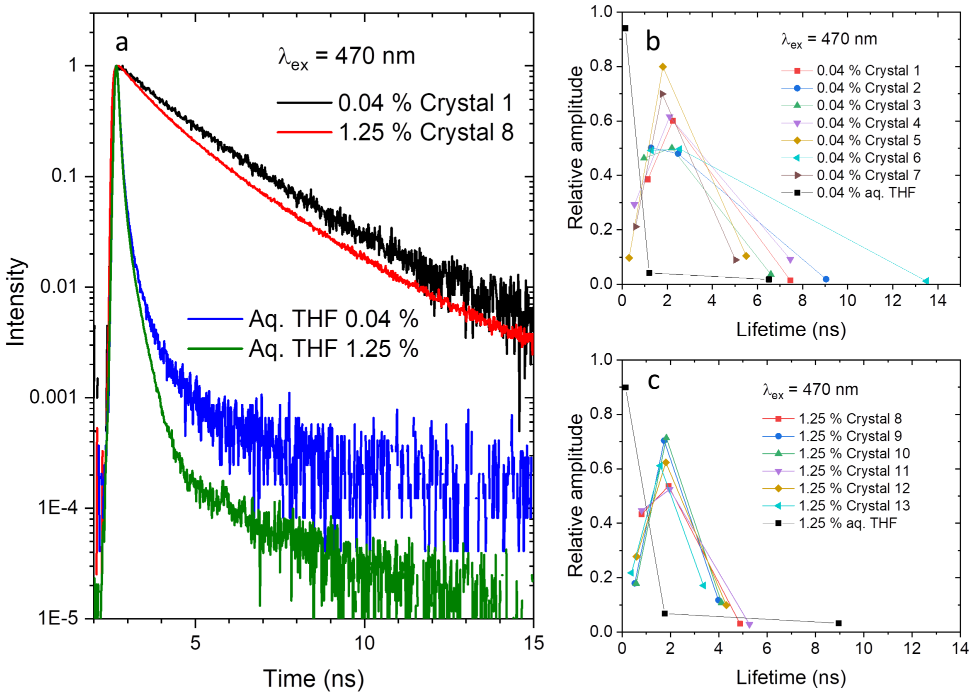


Figure S13: a) Photoluminescence decay curves for DCTC$^{-}$ ($C_2$ peak) in single crystals of doped melamine prepared with mole percentages of TCNQ to melamine of 0.04 % and 1.25 %, in comparison with those for aqueous THF solutions of melamine and TCNQ with mole percentages of TCNQ to melamine of 0.04 % and 1.25 %, measured at a laser wavelength of 470 nm and an emission band of 500 – 550 nm. b) Three lifetimes and their relative amplitudes evaluated for 0.04 % crystals 1–7, and for the 0.04 % precursor solution. c) Three lifetimes and their relative amplitudes evaluated for 1.25 % crystals 8–13, and for the 1.25 % precursor solution.

# References

[1] V. Barone and M. Cossi. Quantum calculation of molecular energies and energy gradients in solution by a conductor solvent model. *The Journal of Physical Chemistry A*, 102(11):1995–2001, 1998.

[2] Frank E. Critchfield, John A. Jr. Gibson, and James L. Hall. Dielectric constant and refractive index from 20 to 35° and density at 25° for the system tetrahydrofuran—water1. *Journal of the American Chemical Society*, 75(23):6044–6045, 1953.
